# Effectiveness of camera traps for quantifying daytime and nighttime visitation by vertebrate pollinators

**DOI:** 10.1002/ece3.4438

**Published:** 2018-08-24

**Authors:** Siegfried L. Krauss, David G. Roberts, Ryan D. Phillips, Caroline Edwards

**Affiliations:** ^1^ Science Directorate Botanic Garden and Parks Authority Kings Park and Botanic Garden Perth Western Australia Australia; ^2^ School of Biological Science The University of Western Australia Crawley Western Australia Australia; ^3^ Centre for Natural Resource Management The University of Western Australia Albany Western Australia Australia; ^4^ Ecology and Evolution Research School of Biology The Australian National University Canberra Australian Capital Territory Australia; ^5^ Department of Ecology, Environment and Evolution La Trobe University Melbourne Victoria Australia; ^6^ Oberlin College Oberlin Ohio USA

**Keywords:** *Banksia*, camera trapping, honey possum, honeyeaters, plant mating, pollination, pollination syndrome, remote sensing, vertebrates

## Abstract

Identification of pollen vectors is a fundamental objective of pollination biology. The foraging and social behavior of these pollinators has profound effects on plant mating, making quantification of their behavior critical for understanding the ecological and evolutionary consequences of different pollinators for the plants they visit. However, accurate quantification of visitation may be problematic, especially for shy animals and/or when the temporal and spatial scale of observation desired is large. Sophisticated heat‐ and movement‐triggered motion‐sensor cameras (“camera trapping”) provide new, underutilized tools to address these challenges. However, to date, there has been no rigorous evaluation of the sampling considerations needed for using camera trapping in pollination research.
We measured the effectiveness of camera trapping for identifying vertebrate visitors and quantifying their visitation rates and foraging behavior on *Banksia menziesii* (Proteaceae). Multiple still cameras (Reconyx HC 500) and a video camera (Little Acorn LTL5210A) were deployed.
From 2,753 recorded visits by vertebrates, we identified five species of nectarivorous honeyeater (Meliphagidae) and the honey possum (Tarsipedidae), with significant variation in the species composition of visitors among inflorescences. Species of floral visitor showed significant variation in their time of peak activity, duration of visits, and numbers of flowers probed per visit. Where multiple cameras were deployed on individual inflorescences, effectiveness of individual still cameras varied from 15% to 86% of all recorded visits. Methodological issues and solutions, and the future uses of camera traps in pollination biology, are discussed.
*Conclusions and wider implications*: Motion‐triggered cameras are promising tools for the quantification of vertebrate visitation and some aspects of behavior on flowers. However, researchers need to be mindful of the variation in effectiveness of individual camera traps in detecting animals. Pollinator studies using camera traps are in their infancy, and the full potential of this developing technology is yet to be realized.

## 1 INTRODUCTION

The use of camera traps in wildlife management, conservation, and research has increased dramatically in recent decades (Burton et al., 2015; Caravaggi et al., 2017; Meek, Ballard, Vernes, & Fleming, 2015; O'Connell, Nichols, & Karanth, 2011; Rovero, Zimmermann, Berzi, & Meek, 2013; Steenweg et al., 2017). Camera traps have primarily been used for faunal surveys, monitoring and population size estimates, as well as species biology and management type issues such as habitat associations, activity patterns, diet, disease monitoring, and monitoring of wildlife crossings (Rovero et al., 2013). Thus far, the vast majority (95%) of 266 camera trapping studies have focused on large mammal species (Burton et al., 2015). Despite their widespread use in many areas of animal ecology, the potential utility of camera traps for studying pollination is only beginning to be realized. Camera traps are likely to be particularly effective in vertebrate pollination systems, which includes members of ca. 500 of the 13,500 vascular plant genera, and more than 1,000 bird, bat, marsupial, rodent and reptile species (Anderson, Kelly, Robertson, & Ladley, 2016; Krauss, Phillips, Karron, Roberts, & Hopper, 2017; Proctor, Yeo, & Lack, 1996). For example, camera traps have recently provided new evidence for rodent pollination (Hobbhahn & Johnson, 2013; Hobbhahn, Steenhuisen, Olsen, Midgely, & Johnson, 2017; Lombardi, Peter, Midgley, & Turner, 2013; Melidonis & Peter, 2015; Zoeller, Steenhuisan, Johnson, & Midgley, 2016), and for detecting promiscuous pollination by flying foxes, sugar gliders, birds, and insects of an Australian baobab (Groffen, Rethus, & Pettigrew, 2016).

In pollination research, camera trapping may be particularly effective at overcoming limitations in the capacity of direct human observation to detect reclusive pollinators. Further, the time for which observations can be made is greatly increased, allowing constant monitoring of flowers over many days, weeks, or even months. Importantly, camera traps also provide untested potential for a detailed quantification of visitation rates and behavior, enabling new insight into the consequences of pollinator behavior for plant mating. The variation in foraging strategies and social behaviors that affect pollinator movements can have profound effects on plant mating (Krauss et al., 2017; Mitchell, Irwin, Flanagan, & Karron, 2009), making quantification of pollinator behavior important for understanding the ecological and evolutionary consequences of different pollinator groups. In this way, camera trap data can be fully utilized to explicitly test ideas or hypotheses, rather than merely estimate abundance or density.

While the potential of camera trapping as a method for pollinator detection is clearly vast, there has been no rigorous evaluation of the sampling considerations needed for this type of study. Such an evaluation will be important for understanding potential issues such as the number of replicate camera traps needed, their effectiveness at detecting different groups of organism, the effect of camera setup (e.g., distance, angle), and ambient conditions (e.g., time of day, temperature), and the data they can reliably collect (Jumeau, Petrod, & Handrich, 2017). Here, we aimed to (a) measure the effectiveness of camera trapping as a method of identifying vertebrate visitors and (b) quantify visitation rates and timing of pollinator visits and (c) resolve which aspects of foraging behavior could be quantified. We focused on *Banksia menziesii* (Proteaceae), a species visited by multiple bird, mammal and insect species, but primarily reliant on vertebrates for pollination (Ramsey, 1988a,b, 1989). Our approach also addresses the recent call (Burton et al., 2015) for more thorough reporting of methodological details to facilitate efforts to evaluate and improve the reliability of camera trapping surveys.

## 2 MATERIALS AND METHODS

### 2.1 Study system

**Figure 1:**
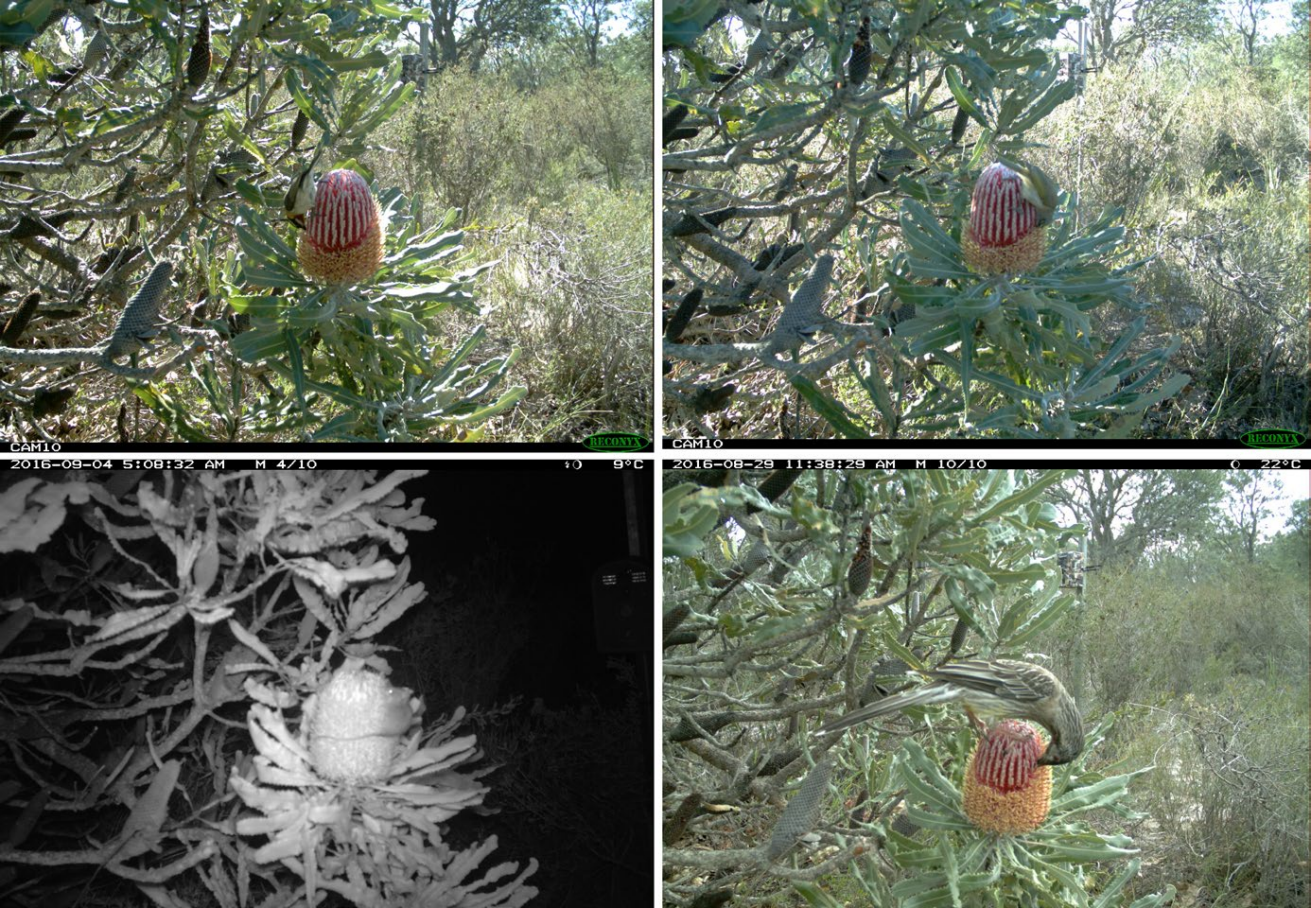
Birds and a honey possum captured by photo traps on *Banksia menziesii* inflorescences, clockwise from top left: male Western Spinebill, Brown Honeyeater, Red Wattlebird, honey possum

*Banksia menziesii* (Proteaceae) is a common tree or woody shrub of *Banksia* woodlands, a threatened ecological community endemic to sandy soils of southern Western Australia (Collins, Collins, & George, 2008). Flowering occurs from February to October with a peak in June. Inflorescences are most commonly red, but yellow or pink variants occur in some populations. Each inflorescence has 600–1,400 nectar‐producing flowers arranged orthogonally around a central woody axis of up to 12 cm in length, with ca. 40–60 flowers open on any single day (Figure 1; Ramsey, 1988a).

*Banksia menziesii* is self‐incompatible, so dependent on pollinators facilitating cross‐pollination for seed set to occur (Ramsey & Vaughton, 1991). Floral visitors to *B. menziesii* that have been documented thus far include the honeyeaters (Meliphagidae), Western Spinebill (*Acanthorhynchus superciliosus*), Red Wattlebird (*Anthochaera carunculata*), Western Wattlebird (*A. lunulata*), Singing Honeyeater (*Lichenostomus virescens*), Brown Honeyeater (*Lichmera indistincta*), White‐cheeked Honeyeater (*Phylidonyris nigra*), and the New‐Holland Honeyeater (*P. novaehollandiae*), as well as the silvereye (*Zosterops lateralis*), honey possum (*Tarsipes rostratus*), staphylinid beetles (Coleoptera, Staphylinidae), European Honey‐bee (*Apis mellifera*) and native bees in the genera *Hylaeus* and *Leioproctus* (Brown et al., 1997; Houston, 2000; Ramsey, 1988b, 1989).

### 2.2 Camera trapping

Camera trapping was undertaken within the ca. 1,200 Ha Ioppolo Nature Reserve (INR), a relatively pristine *Banksia* woodland remnant located 65 km north of Perth, Western Australia (31°29′S, 115°57′E). *B. menziesii*, along with the summer flowering *B. attenuata*, are dominant members of the overstory at INR. From 22 June 2016 to 14 October 2016 (114 trapping days and nights), motion‐triggered cameras were set up to record all visits by vertebrates to 12 inflorescences on nine plants, located within a one‐hectare area. For still images, we used the RECONYX Hyperfire HC500 (http://www.reconyx.com/product/HC500-HyperFire-High-Output-Covert-IR), which is a mid‐price‐range camera capable of detecting small animals. This camera has an image resolution of 1080P high definition and passive infrared sensor to detect a differential in heat‐and‐motion between a subject and the background temperature, and a “low‐glow” infrared flash array. Recorded temperatures ranged from −3 to 32°C, with a mean of 18°C. Cameras were mounted at varying heights up to 1.5 m on star pickets using zip ties and positioned at the same height as the inflorescence approximately 60 cm away (Figure 1). Inflorescences just beginning to bloom were arbitrarily chosen for monitoring so as to collect visitation data throughout the entire life‐span of each inflorescence.

Camera settings were as follows: sensitivity = high; pictures per trigger = 10; picture interval = rapid‐fire; quiet period = no delay. These settings armed the camera to take 10 photos over ca. 9 s when triggered by motion, with a trigger speed of 1/5th second. The cameras continue to capture bursts of photographs as long as there is movement detected, and capture images day and night. On average, cameras (or batteries – we used rechargeable AA Panasonic eneloop batteries) were changed every 2 weeks, and digitally stored photographs downloaded to a computer. Overall, 20 cameras were used (on some inflorescences we employed multiple cameras – see below), and dates and location of cameras and inflorescences recorded.

Downloaded photographs were scored manually for the presence of vertebrate visitors to inflorescences. Individual photographs were imprinted with date, time, photograph number in the series of 10, temperature, and camera number. For each visit captured by cameras, species, date, start time, finish time, duration of visit, number of flowers probed, inflorescence flowering stage, and temperature, were recorded. Inflorescence flowering stage identifies the cumulative proportion of flowers that have opened on an inflorescence, so, for example, a proportion of 0.1 indicates that approximately 10% of flowers have opened from the base of the inflorescence. For Western Spinebills, males and females were distinguished by clear differences in plumage. When a visit was longer than one series of 10 photographs (ca 9 s), duration of visit was estimated from the arrival time (typically photo 1 of 10) and then the time of departure, which often included a short but variable lag period (typically 2–30 s) between the final photograph in the first series and the first triggered photograph of the next series. We tested for variation in visitor composition within and among inflorescences with χ^2^ tests using SYSTAT v13 software. The number of probes was estimated from photographs and contrasted to accurate estimates of probe rate obtained from additional video camera footage (see below). Differences in the mean duration of visits by each bird species to inflorescences were assessed by one‐way Analysis of Variance and post hoc Tukey tests using SYSTAT v13 software.

### 2.3 Quantification of camera effectiveness in visitor detection

At most inflorescences, multiple cameras (2, 3 or 4) were deployed at equal distances (60 cm) from the same side of an inflorescence. This overlap in monitoring enabled an assessment of the accuracy of cameras based on the number of known visits that went undetected by a given camera. Data from multiple cameras on the three inflorescences with the greatest overlap in recording was assessed to generate a relative effectiveness index for each camera, calculated as the number of visits captured by a single camera divided by the total number of visitors captured across all cameras at that inflorescence, and multiplied by 100 to convert it to relative percentage effectiveness. Thus, relative effectiveness index measures the percentage of known visits recorded by a single camera.

**Figure 2:**
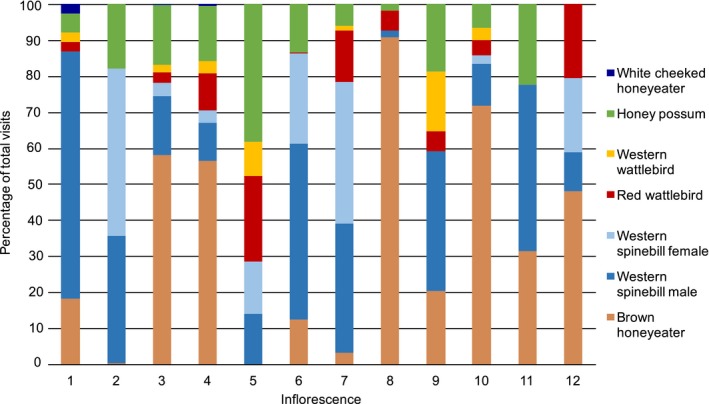
The relative percentage of total visits for each vertebrate visitor for each of 12 *Banksia menziesii* inflorescences as captured by camera traps

To compare the effectiveness of still photos with video‐based camera trapping, still photographs were complemented with motion‐triggered videos shot with a LITTLE ACORN LTL5210A 12‐mega pixel scouting camera (http://ltlacorn.com.au) set to shoot 60 s videos once triggered. In total, we recorded 97.6 min from 172 visits across three inflorescences (numbered 2,3 and 10; Figure 2) between 20/7/16 – 9/8/16, 15/8/16 – 18/8/16, and 30/9/16 – 5/10/2016, respectively. For each video, date, start time, finish time, duration of visit, number of floral probes and the species visiting was recorded. Mean visit length and mean number of floral probes per species per second was estimated. When videos and cameras captured the same visit, we contrasted duration of visit and mean probe rate for all birds to assess whether duration and probe rates captured by cameras underestimated the values as determined from videos, and assessed the significance of differences by dependent *t*‐tests for paired samples using SYSTAT v13 software.

## 3 RESULTS

### 3.1 Quantification of visitors

In total, the still cameras monitored 10,272 hr over 15 weeks. Across the 12 inflorescences of *B. menziesii* monitored, 2,753 visits by vertebrates were recorded. For example, from one camera we retrieved 11,500 photos from 597 different visits over the full 25 days of an inflorescence flowering. Almost all (99.3%) visitors could be identified to species level. The percentage of total visits for each species were Western Spinebill (45%), Brown Honeyeater (34%), Red Wattlebird (4%), Western Wattlebird (2%) and White‐cheeked Honeyeater (<1%), and the honey possum (15%) (Figure 1). Other than nectarivores, one visit of a female Red‐capped Robin (*Petroica goodenovii*) perching, but not feeding, on top of a *B. menziesii* inflorescence was recorded.

Relative visitation percentages varied significantly among inflorescences (χ^2 ^= 1,788, *df* = 66, *p *<* *0.001; Figure 2). Of the most common visitors, Brown Honeyeater visits as a percentage of total visits varied from 0% for Inflorescence 2 to 91% for Inflorescence 8, male Western Spinebills varied from 0.02% (Inflorescence 8) to 68% (Inflorescence 1), and female Western Spinebills varied from 0% (for 4 Inflorescences) to 46% for Inflorescence 2 (Figure 2). Relative percentage visits by male and female Western Spinebills often varied substantially on the same inflorescence (e.g., 68% and 0%, respectively, for Inflorescence 1).

### 3.2 When are vertebrates visiting?

**Figure 3:**
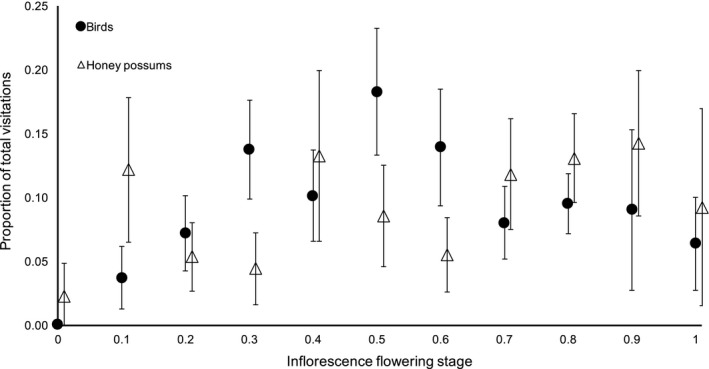
The mean (±*SE*) proportion of total bird (*n* = 1,911) and honey possum (*n* = 353) visits that occurred at each of 10 flowering stages for 5 inflorescences of *Banksia menziesii* that were monitored over their entire duration. The inflorescence flowering stage is ranked from 0 to 1, where, for example, 0 indicates no flowers opened and 0.5 represents 50% of the inflorescence has flowered

Visitation by birds and honey possums was not independent of inflorescence flowering stage (birds, χ^2^ = 839, *p *<* *0.001, *df* = 36; honey possums, χ^2^ = 105, *p *<* *0.001, *df* = 16 (data from three inflorescences were combined due to low numbers in many cells); Figure 3). On individual inflorescences, bird visits increased gradually to a maximum visitation rate (18% of total visits) at mid‐inflorescence flowering (i.e., when 50% of the flowers had opened), and declined gradually from there (Figure 3). The variation in honey possum visitation with inflorescence flowering stage was more idiosyncratic, with no clear trend over time (Figure 3). Honey possum visits were also recorded before and after flowering (presumably when pollen and nectar production had ceased) (Figure 3). Cameras also commonly captured honey possums foraging widely across inflorescences including above and below the currently opened flowers, while birds were foraging at the advancing front of open flowers in >99% of recorded visits.

**Figure 4:**
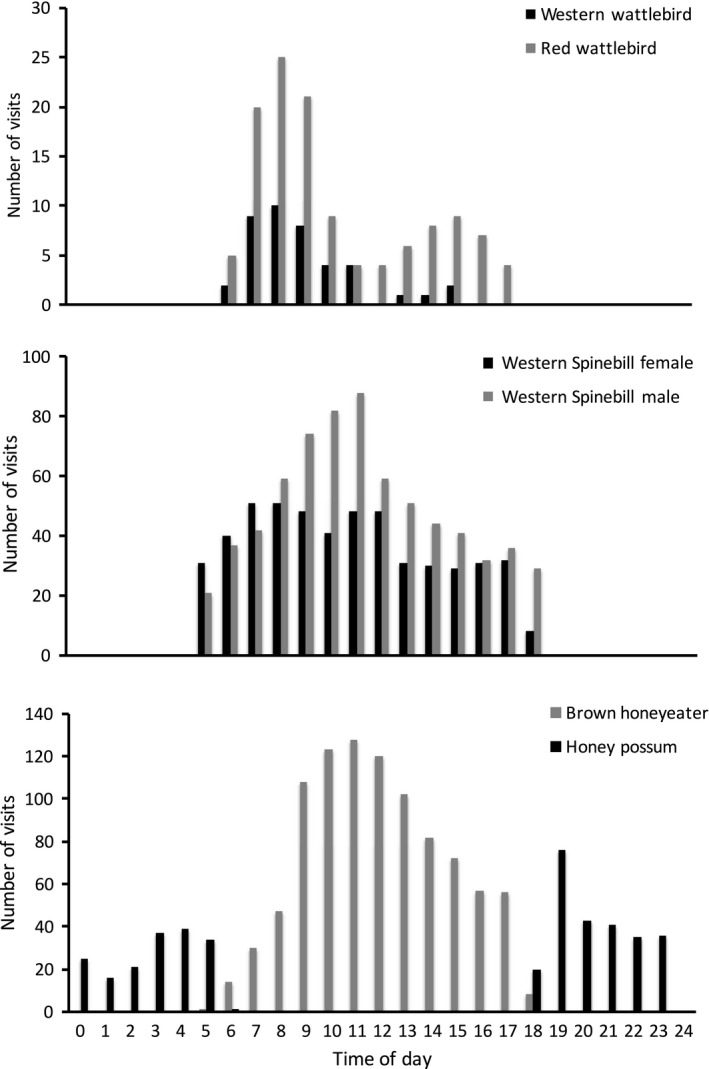
Frequency of recorded visits to *Banksia menziesii* inflorescences by each vertebrate species per hour by time of day (24 hr clock). All bird visits were diurnal, all honey possum visits were nocturnal. *Y*‐axis scales differ among plots

Although there was no time overlap between (diurnal) bird and (nocturnal) honey possum visits, we did record a first diurnal bird visit on one inflorescence that was only 12 min after a honey possum visit. Bird visitation occurred throughout the day, but patterns of activity varied across species (Figure 4). For example, visitation peaked late morning (11 a.m.) for Brown Honeyeaters and Western Spinebill males, and early morning (8 a.m.) for both species of wattlebirds (Figure 4). Western Spinebill females did not show a peak visitation time, rather a steady visitation rate per hour through the morning, which was again steady but lower through the afternoon. Honey possums were recorded throughout the night, with a peak in the hour after sunset (Figure 4).

### 3.3 The behavior of floral visitors

Only three intra‐ or interspecies aggressive interactions between birds were recorded. All vertebrate visitors were recorded probing flowers and therefore assumed to transfer pollen. Mean recorded duration of visits by birds to inflorescences differed among species (ANOVA; *F *=* *29.7; *p *<* *0.001; *df* = 4 (note White‐cheeked Honeyeater was excluded from the analysis due to too few data (*N* = 4)). From still photos, the mean (±*SE*;* N*) recorded duration of visit to an inflorescence per species was significantly greater (*p *<* *0.01) for Red Wattlebirds (42 s ± 4.0; 117) than all other birds. Brown Honeyeater mean (±*SE*;* N*) duration (26.1 s ± 0.9; 921) was significantly greater (*p *<* *0.01) than Western Spinebill Males (17.8 s ± 0.8; 688) and Western Spinebill Females (18.7 s ± 0.9; 513) but not Western Wattlebirds (22.0 s ± 3.0; 46). All other comparisons were not significantly different. Video footage of 172 bird visits (97% of which were Western Spinebills or Brown Honeyeaters) showed that different flowers on a single inflorescence were probed on average once every 3.3 s during a visit. For the most common Western Spinebills and Brown Honeyeaters, variation in probe rate among inflorescences per species was greater than the variation among species (Table 1). Across all video recorded visits by birds (*N* = 172), the overall mean (±*SE*) length of visit was 34 (±1.6)s, and the mean (±*SE*) number of floral probes per visit was 8.6 (±0.3), with a range of 1–21.

**Table 1:** Floral probe rates recorded by motion‐triggered videos of bird visitors to three inflorescences (labelled 2, 10 and 11) of *Banksia menziesii*

Inflorescence	Species	Mean (+/‐ standard error) probes/s	Number of s/floral probe	Number of visits recorded	Total time recorded (s)
2	Western Spinebill male	0.21 (0.03)	4.81	16	696
2	Western Spinebill female	0.25 (0.02)	3.97	34	1,242
11	Western Spinebill male	0.42 (0.02)	2.37	33	830
11	Brown honeyeater	0.34 (0.03)	2.93	15	333
10	Brown honeyeater	0.27 (0.01)	3.71	67	2,603
10	Western Spinebill male	0.34 (0.17)	2.94	2	44
10	Red wattlebird	0.21 (0.05)	4.67	2	32
10	Western wattlebird	0.40 (0.06)	2.50	3	79
Overall		0.30 (0.01)	3.34	172	5,859

### 3.4 Camera trapping effectiveness

**Figure 5:**
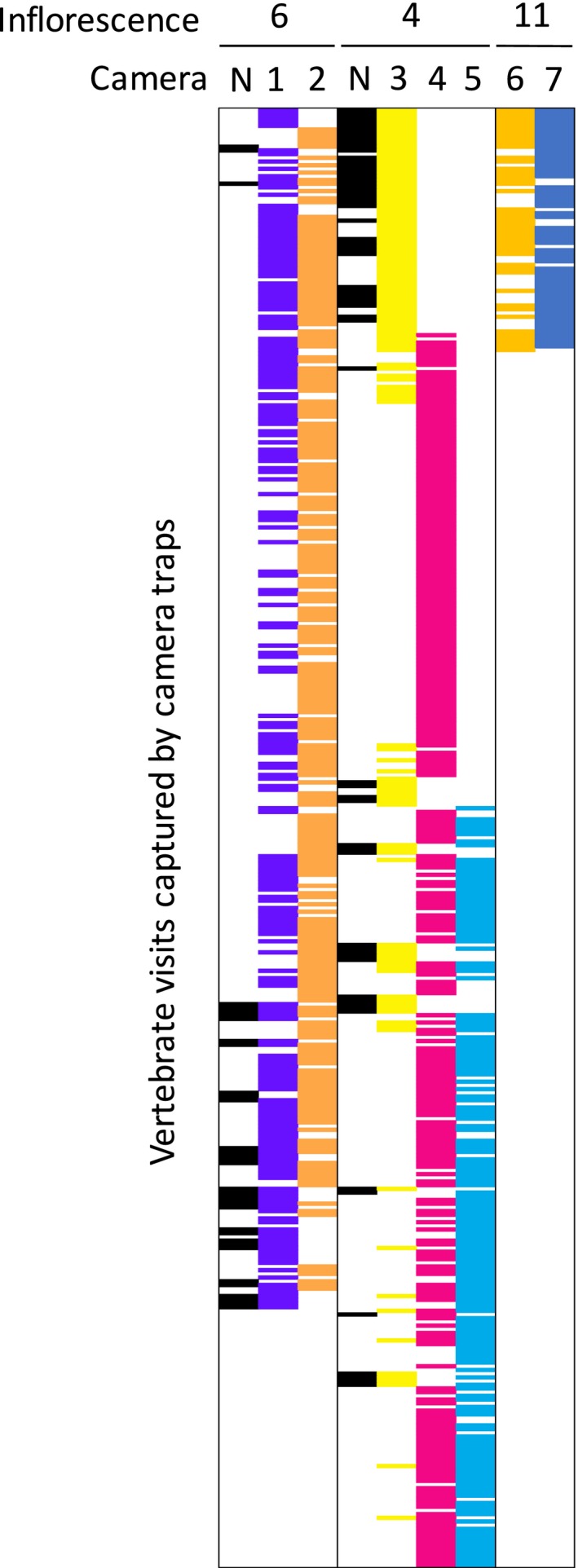
Diagrammatic representation of recorded vertebrate visits for multiple cameras on each of three *Banksia menziesii* inflorescences. Figure demonstrates relative effectiveness of each camera, where nonoverlapping bars show failure of one or more cameras to detect a visit recorded by at least one camera. Black indicates a nocturnal honey possum visit

Mean (±*SE*) camera effectiveness was 62.4 (±7.5)%, the median (and interquartile range) were (54%‐) 63.9% (−76%), and range 15%–86%. The most effective camera recorded 597 visits out of a total of 695 visits recorded from all cameras across 25 flowering days of a single inflorescence. The 15% effective camera was an outlier, as the next lowest camera was 50% effective. The 15% effective camera (camera 3 on inflorescence 4) also performed much better at night than the other two cameras stationed at the same inflorescence (20 of 25 night visits recorded only by camera 3), but performed poorly during the day (35 of 311 day visits recorded by camera 3; Figure 5). In contrast, one of these cameras (camera 4 on inflorescence 4) failed to detect any visits during the night (Figure 5).

For all bird visits captured by both still and video cameras on 3 inflorescences (*N* = 110), cameras underestimated the length of visit (mean [±*SE*] for camera = 28.0 (2.5)s vs video = 36.7 (1.9)s, respectively; *t *=* *2.83; *p *=* *0.002; *df* = 109). However, there was no difference in the detection of floral probe rate (mean [±*SE*] for camera = 0.33 (0.01) vs video = 0.32 (0.01) probes per sec, respectively; *t *=* *0.39; *p *=* *0.34; *df* = 109). That is, the rate at which birds probed flowers while visiting inflorescences was accurately estimated from photos generated by our camera traps.

## 4 DISCUSSION

### 4.1 Detecting pollinator species and their behavior

Five species of honeyeater and the honey possum were confirmed visitors to *B. menziesii* flowers in INR by camera traps. Western Spinebills and Brown Honeyeaters accounted for 79% of 2,753 vertebrate visits recorded*,* which was nearly identical (82% of 923 observations) to an earlier observational study in similar *Banksia* woodland (Newland & Wooller, 1985), but contrasted to another study that found Brown Honeyeaters, New Holland Honeyeaters, and Western Wattlebirds were frequent visitors to *B. menziesii*, with the Western Spinebill and Red Wattlebird relatively uncommon (Ramsey, 1989). Our camera traps showed that honeyeater visits to inflorescences occurred throughout the day but peaked mid‐ to late‐morning, lasted on average 34 s during which an average of 8.6 flowers was probed, strongly suggesting they all contribute to effecting pollen transfer, much of it within inflorescences. Pollinator exclusion experiments confirm that these honeyeaters are effective floral visitors for pollen removal and deposition on stigmas (Ramsey, 1988b). Combined, these results highlight that the importance of different honeyeater species for *B. menziesii* is likely to vary within and between sites depending on the composition of the local honeyeater community.

After the Western Spinebill and Brown Honeyeater, honey possums were the next frequent visitor to flowers of *B. menziesii* at INR, accounting for 15% of all recorded visits, which occurred throughout the night, with a peak during the hour after sunset. Due to the low diversity of co‐flowering food plants, *B. menziesii* is expected to be the most important source of pollen and nectar for honey possums in INR during the winter and spring months. Given the behavior and frequency of visitation recorded here, where many photos showed honey possums with snouts deeply buried in inflorescences, we predict that they play a significant role in pollen transfer in *B. menziesii*, possibly predominantly geitonogamous. Based on heavy pollen loads on heads and snouts, honey possums have been inferred to be an important pollinator of several species of *Banksia* (Bradshaw et al., 2007; Wiens, Renfrew, & Wooller, 1979; Wooller & Wooller, 2013).

### 4.2 Methodological issues – quantifying behavior

Although camera traps can disturb the natural behavior of some animals (Glen, Cockburn, Nichols, Ekanayake, & Warburton, 2013; Meek et al., 2014) there was little evidence to suggest that our camera traps affected the behavior of birds and honey possums. In our study, average visits of 34 s by honeyeaters, and recorded visits of several minutes by honey possums, suggest that most individuals are not obviously affected by cameras, even in low light when the infrared flash was activated. We also generated numerous videos of up to 60 s where vertebrate visitors were clearly unaffected by the activation of the camera flash.

Our extra video footage was a critical supplement to photos for assessment of still cameras and documenting visitor behavior. While probe rate was accurately recorded by still cameras, time spent on inflorescences, and therefore number of floral probes per visit, were underestimated compared to video. Video data is much more memory intensive, and the capacity of memory cards was a limiting factor to the potential recording life – in some cases, capacity was filled after only 3 days of recording. However, videos are undoubtedly more informative than still images, and will be increasingly utilized for camera trapping as the technology continues to develop and costs decrease (Caravaggi et al., 2017). For our purposes, videos of 60 s duration nearly always captured an entire visit by birds to an inflorescence.

An important limitation of both video and photograph‐based camera trapping is the ability to detect aggression between individuals, a characteristic of nectarivorous honeyeaters that leads to frequent disruption to foraging (e.g., Phillips, Steinmeyer, Menz, Erickson, & Dixon, 2014). Aggression often takes the form of prolonged pursuits, suggesting that camera trapping is unlikely to replace direct observation for understanding the significance of aggression for pollination and pollen dispersal.

### 4.3 Methodological issues ‐ technical

Despite significant advances in the quality of camera traps (Rovero et al., 2013), the use of camera trapping to accurately quantify behavior and visitation rates of vertebrate pollinators poses significant challenges. The ultimate scenario is one where each camera captures the entire duration of every visit during the day and night. Clearly, the cameras we employed fell short of this objective, despite identical settings on the same model of camera similarly positioned relative to the inflorescence. Detection at night in particular was highly variable among cameras. Consequently, multiple cameras were necessary for accurate quantification of visitation rates. Routine cleaning of the infrared detection array window, including the mask, lens and light meter, may improve the effectiveness of cameras, as is the routine use of a moisture‐absorbing desiccant system within the camera housing.

False triggers, where the camera was triggered despite no visitor, was a significant issue that varied depending on camera, weather conditions and physical setup. False triggers were typically a result of vegetation movement caused by wind, particularly on sunny days. Here, as leaves warm, cameras cannot distinguish between warmed leaves moving with the wind and warm‐blooded animals moving in the scene. In some cases, as many as 99% of many thousands of photos were generated by false triggers for individual cameras, inefficiently consuming memory and battery power, and necessitating a time‐consuming screening process of elimination. Choosing relatively sheltered inflorescences that were up to one meter above the ground, and avoiding the sun shining directly on the face of the camera, appeared to help minimize false triggers. At the other extreme, detection lags or failures impacted effectiveness and the accuracy of estimates of the length of pollinator visits, resulting in gaps of 2–30 (‐ 60) seconds between multiple series of photos of the same visit. Color marking of birds to enable the recognition of individuals in photos would help to address this issue.

Other aspects of the camera trapping method demonstrated excellent performance. The quality of photos was such that all vertebrate species could be identified. With 12 AA rechargeable batteries per camera, camera life is claimed to be up to 40,000 images, so cameras can be left in the field for many weeks or even months, as long as the memory card has sufficient capacity (32GB is possible, our photos were ca 200–700 KB each). The use of an external solar power pack can overcome the finite power capacity of conventional batteries. Critically, camera trap photos and videos provide an on‐going resource library of objective visitation data that can be returned to at any time for checking and/or extracting additional information, a clear advantage over field observational data.

Rovero et al. (2013) provide a review of multiple cameras (see their Table 2) split into high‐end ($550–$1,000), mid range (ca $450), and low end (ca $200). We used a mid‐range camera, and it may be that high‐end cameras have better effectiveness than these mid‐range cameras, and where more accurate quantification of the number of visitors is required, it might be necessary to invest in these more expensive cameras. For our purposes, having multiple cameras on each inflorescence was critical, potentially leading to a higher cost per inflorescence than one superior camera. As such, before larger scale implementation of camera trapping of floral visitors, further testing of other models is required.

One option for collecting visitation data that does not rely on the ability of the camera to detect movement is to utilize cameras with time‐lapse capacity, in a similar way to phenocam networks that are monitoring vegetation status and environmental changes (Brown et al., 2016; https://phenocam.org.au). For example, a time‐lapse of one photo every ten seconds would capture almost all visits in our system without the triggering requirement. At that rate, 8,640 pictures would be captured daily, then screened to find photos of visits and data recorded. While this would standardize all the cameras and could produce a more reliable data set, there is a cost involved in the screening of photos. Here, efficient tools to manage camera trap data, such automated image recognition software, are critical (e.g., Jumeau et al., 2017; Niedballa, Sollmann, Courtiol, & Wilting, 2016; Tack et al., 2016), and citizen science can assist (e.g., McShea, Forrester, Costello, He, & Kays, 2016; https://www.zooniverse.org/projects/birgus2/western-shield-camera-watch/classify). Increasingly sophisticated digital video recording devices (e.g., GoPro, https://gopro.com) offer another alternative and/or complementary option to still cameras that have enormous potential for, and are beginning to be applied to, pollination biology studies, even with invertebrates (e.g., Gilpin, Denham, & Ayre, 2017).

### 4.4 Future uses of camera traps

The strengths of camera traps as a methodology for pollination biology lie with their ability to detect shy floral visitors, to continuously monitor multiple plants for an extended period of time, and to dramatically increase the number of visits recorded. From both theoretical and conservation perspectives, there are several areas where this advance could make a significant contribution. In particular, camera trapping provides an effective way of detecting floral visitors when testing the predictions of pollination syndromes. Pollination syndromes are suites of floral traits that are often associated with particular groups of pollinators (Rosas‐Guerrero et al., 2014). In many cases, pollination syndromes have strong predictive power (Johnson & Wester, 2017). However, exceptions occur even in well‐supported syndromes (e.g., Quintero, Genzoni, Mann, Nuttman, & Anderson, 2017), and clear associations between pollinator groups and floral traits are not evident in some plant communities or taxonomic groups (Ollerton et al., 2009), particularly those with more generalist species. As such, pollination syndromes should be considered working hypotheses until tested, and camera traps provide an efficient tool to test syndrome predictions. Following detection of vertebrate pollinators with camera traps, experiments and/or quantification of pollen loads are critical to confirm the effectiveness of the floral visitor (e.g., Ramsey, 1988b).

Camera traps assessing pollination extend their use from a conservation perspective (Caravaggi et al., 2017). Globally, there is clear evidence of recent declines in bird and mammal pollinators (Potts et al., 2010; Regan et al., 2015). For example, South Western Australia is a Global Biodiversity Hotspot where ca. 15% of 8,379 native vascular plant taxa (Gioia & Hopper, 2017) and ca. 40% of species listed as threatened flora are pollinated by vertebrates (Brown et al., 1997; Keighery, 1982). Here, several species of nectarivorous vertebrates are experiencing population decline in landscapes negatively impacted by land clearing, habitat fragmentation, introduced species, climate change and/or disease (Davis, Gole, & Roberts, 2013; Davis et al., 2014; How & Dell, 2000; Phillips, Hopper, & Dixon, 2010). Camera trapping can make a positive contribution to conservation and ecological restoration by identifying and quantifying floral visitors, documenting decline in abundance or local extinction, documenting changes in behavior, detecting them in candidate sites for conservation translocations, and/or the detection of possible replacement pollinators.

A significant growth area in the field of pollination biology is the collection of data across entire plant‐pollinator communities to investigate issues such as differences in specialization between communities, mechanism of species co‐existence, how communities are structured, and the role of diversity in community resilience (e.g., Aizen, Sabatino, & Tylianakis, 2012; Pauw & Stanaway, 2015). However, these studies present the challenge of not only detecting pollinators for a large number of plant species, but also detecting enough visits to enable an accurate estimate of specialization, as plants with only a few recorded visits can only have a few pollinator species detected (Bluthgen, 2010). For species with low visitation rates, camera trapping provides the potential to drastically increase the number of floral visitors observed and avoid the bias toward specialization due to small sample sizes. As such, at least for communities of vertebrates, camera trapping has the potential to make an important contribution to understanding the mechanisms underpinning community structure.

Camera traps document visitation but not visitor movements to and from an inflorescence. However, knowledge of interflower movements within and among plants is critical for an understanding of the consequences of visitor behavior on plant mating (Krauss et al., 2009; Krauss et al., 2017). Observational studies could be optimized from preliminary camera trapping to determine the period of peak pollinator activity. A powerful complement to observational studies is to employ an extensive network of camera traps synchronized for time and date, with banded birds so that individuals, rather than just species, can be identified on camera images. In this way, movement maps can be constructed for individuals based on time, date and location from photos or videos documenting visits.

## 5 CONCLUSIONS

Our study has demonstrated that camera trapping is an exceptional tool for pollination biology studies that not only seek to identify vertebrate visitors, but also to quantify some aspects of behavior such as visitation patterns. In this way, camera traps provide a powerful addition to observation, especially when complemented with individual bird identification through banding, the use of trackers to document movement, and genetic markers for paternity assignment to document realized pollen dispersal and paternal diversity within and among fruits. However, given the inconsistency between the cameras we used, multiple cameras on individual flowers/inflorescences/plants are recommended, and these could be complemented by motion‐triggered digital video recorders and/or time‐lapse photography for further detail on visitation behavior. Increasingly, sophisticated cameras employing time‐lapse photography perhaps currently provide the most powerful capacity for accurate quantification of visitation by vertebrates at flowers, although even these bring their own set of challenges that include prolonged data scoring and a reduced ability to quantify behavior. Pollinator studies using camera traps are in their infancy, and the full potential of this developing technology is yet to be realized. These new tools offer exciting new insights into potentially novel ecological and evolutionary consequences for plants pollinated by vertebrates.

## CONFLICT OF INTEREST

Authors declare no conflict of interest.

## AUTHORS’ CONTRIBUTIONS

SLK and DGR conceived and executed the study and collected the data; SLK and CE analyzed the data; SLK led the writing of the manuscript. All authors contributed significantly to the drafts and gave final approval for publication.

## DATA ACCESSIBILITY

Data available from the Dryad digital repository https://doi.org/10.5061/dryad.tj7pp1b

## References

[ece34438-bib-0001] Aizen, M. A. , Sabatino, M. , & Tylianakis, J. M. (2012). Specialization and rarity predict nonrandom loss of interactions from mutualist networks. Science, 335, 1486–1489. 10.1126/science.1215320 22442482

[ece34438-bib-0002] Anderson, S. H. , Kelly, D. , Robertson, A. W. , & Ladley, J. J. (2016). Pollination by birds, a functional analysis In Sekercioglu C. H. (Ed.), Why birds matter: Avian ecological function and ecosystem services (pp. 73–106). Chicago, IL: University of Chicago Press.

[ece34438-bib-0003] Bluthgen, N. (2010). Why network analysis is often disconnected from community ecology: A critique and an ecologist's guide. Basic and Applied Ecology, 11, 185–195. 10.1016/j.baae.2010.01.001

[ece34438-bib-0004] Bradshaw, S. D. , Phillips, R. , Tomlinson, S. , Holley, R. , Jennings, S. , & Bradshaw, F. J. (2007). Ecology of the Honey possum, *Tarsipes rostratus*, in Scott National park, Western Australia. Australian Mammalogy, 29, 25–38. 10.1071/AM07003

[ece34438-bib-0005] Brown, E. M. , Burbidge, A. H. , Dell, J. , Edinger, D. , Hopper, S. D. , & Wills, R. T. (1997). Pollination in Western Australia: A Database of animals visiting flowers. Handbook No. 15, ‐Western Australian Naturalists’ Club, Perth, Western Australia.

[ece34438-bib-0006] Brown, T. B. , Hultine, K. R. , Steltzer, H. , Denny, E. G. , Denslow, M. W. , Granados, J. , … Richardson, A. D. (2016). Using phenocams to monitor our changing Earth: Toward a global phenocam network. Frontiers in Ecology and the Environment, 14, 84–93. 10.1002/fee.1222

[ece34438-bib-0007] Burton, A. C. , Neilson, E. , Moreira, D. , Ladle, A. , Steenweg, R. , Fisher, J. T. , … Boutin, S. (2015). Wildlife camera trapping: A review and recommendations for linking surveys to ecological processes. Journal of Applied Ecology, 52, 675–685. 10.1111/1365-2664.12432

[ece34438-bib-0008] Caravaggi, A. , Banks, P. B. , Burton, A. C. , Finlay, C. M. V. , Haswell, P. M. , Hayward, M. W. , … Wood, M. D. (2017). A review of camera trapping for conservation behaviour research. Remote Sensing in Ecology and Conservation, 3, 109–122. 10.1002/rse2.48

[ece34438-bib-0009] Collins, K. , Collins, K. , & George, A. (2008). Banksias. Melbourne, Vic: Bloomings Books.

[ece34438-bib-0010] Davis, R. A. , Gole, C. , & Roberts, J. D. (2013). Impacts of urbanisation on the native avifauna of Perth, Western Australia. Urban Ecosystems, 16, 427–452. 10.1007/s11252-012-0275-y

[ece34438-bib-0011] Davis, R. A. , Valentine, L. E. , Craig, M. D. , Wilson, B. , Bancroft, W. J. , & Mallie, M. (2014). Impact of *Phytophthora*‐dieback on birds in *Banksia* woodlands in south west Western Australia. Biological Conservation, 171, 136–144. 10.1016/j.biocon.2014.01.027

[ece34438-bib-0012] Gilpin, A. M. , Denham, A. J. , & Ayre, D. J. (2017). The use of digital video recorders in pollination biology. Ecological Entomology, 42, 383–388. 10.1111/een.12394

[ece34438-bib-0013] Gioia, P. , & Hopper, S. D. (2017). A new phytogeographic map for the Southwestern Australian Floristic Region after an exceptional decade of collection and discovery. Botanical Journal of the Linnean Society, 184, 1–15. 10.1093/botlinnean/box010

[ece34438-bib-0014] Glen, A. S. , Cockburn, S. , Nichols, M. , Ekanayake, J. , & Warburton, B. (2013). Optimising camera traps for monitoring small mammals. PLoS ONE, 8, e67940 10.1371/journal.pone.0067940 23840790 PMC3695914

[ece34438-bib-0015] Groffen, J. , Rethus, G. , & Pettigrew, J. (2016). Promiscuous pollination of Australia's baobab, the boab, *Adansonia gregorii* . Australian Journal of Botany, 64, 678–686. 10.1071/BT16049

[ece34438-bib-0016] Hobbhahn, N. , & Johnson, S. D. (2013). A new record of rodent pollination in the holoparasitic genus *Cytinus* . South+A1443 African Journal of Botany, 86, 168 10.1016/j.sajb.2013.02.112

[ece34438-bib-0017] Hobbhahn, N. , Steenhuisen, S. L. , Olsen, T. , Midgely, J. J. , & Johnson, S. D. (2017). Pollination and breeding system of the enigmatic South African parasitic plant *Mystropetalon thomii* (Mystropetalaceae): Rodents welcome, but not needed. Plant Biology, 19, 775–786. 10.1111/plb.12580 28504871

[ece34438-bib-0018] Houston, T. F. (2000). Native bees on wildflowers in Western Australia. Perth, WA: Western Australian Insect Study Society Inc.

[ece34438-bib-0019] How, R. A. , & Dell, J. (2000). Ground vertebrate fauna of Perth's vegetation remnants: Impacts of 170 years of urbanization. Pacific Conservation Biology, 6, 198–213. 10.1071/PC000198

[ece34438-bib-0020] Johnson, S. D. , & Wester, P. (2017). Stefan Vogel's analysis of floral syndromes in the South African flora: An appraisal based on 60 years of pollination studies. Flora, 232, 200–206. 10.1016/j.flora.2017.02.005

[ece34438-bib-0021] Jumeau, J. , Petrod, L. , & Handrich, Y. (2017). A comparison of camera trap and permanent recording video camera efficiency in wildlife underpasses. Ecology and Evolution, 7, 7399–7407. 10.1002/ece3.3149 28944025 PMC5606868

[ece34438-bib-0022] Keighery, G. J. (1982). Bird‐pollinated plants in Western Australia In Armstrong J. A., Powell J. M. & Richards A. J. (Eds.), Pollination and evolution (pp. 77–90). Sydney, NSW: Royal Botanic Gardens.

[ece34438-bib-0023] Krauss, S. L. , He, T. H. , Barrett, L. G. , Lamont, B. B. , Enright, N. J. , Miller, B. P. , & Hanley, M. E. (2009). Contrasting impacts of pollen and seed dispersal on spatial genetic structure in the vertebrate‐pollinated *Banksia hookeriana* . Heredity, 102, 274–285. 10.1038/hdy.2008.118 19002205

[ece34438-bib-0024] Krauss, S. L. , Phillips, R. D. , Karron, J. D. , Roberts, D. G. , & Hopper, S. D. (2017). Novel consequences of bird pollination for plant mating. Trends in Plant Science, 22(5), 395–410. 10.1016/j.tplants.2017.03.005 28412035

[ece34438-bib-0025] Lombardi, G. , Peter, C. , Midgley, J. J. , & Turner, R. (2013). Evidence for rodent‐pollination in *Erica lanuginosa* (Ericaceae). South African Journal of Botany, 86, 175–176. 10.1016/j.sajb.2013.02.138

[ece34438-bib-0026] McShea, W. J. T. , Forrester, T. , Costello, R. , He, Z. , & Kays, R. (2016). Volunteer‐run cameras as distributed sensors for macrosystem mammal research. Biodiversity and Conservation, 31, 55–66.

[ece34438-bib-0027] Meek, P. D. , Ballard, G. A. , Fleming, P. J. S. , Schaefer, M. , Williams, W. , & Falzon, G. (2014). Camera traps can be heard and seen by animals. PLoS ONE, 9, e110832 10.1371/journal.pone.0110832 25354356 PMC4212972

[ece34438-bib-0028] Meek, P. D. , Ballard, G. A. , Vernes, K. , & Fleming, P. J. S. (2015). The history of wildlife camera trapping as a survey tool in Australia. Australian Mammalogy, 37, 1–12. 10.1071/AM14021

[ece34438-bib-0029] Melidonis, C. A. , & Peter, C. I. (2015). Diurnal pollination, primarily by a single species of rodent, documented in *Protea foliosa* using modified camera traps. South African Journal of Botany, 97, 9–15. 10.1016/j.sajb.2014.12.009

[ece34438-bib-0030] Mitchell, R. J. , Irwin, R. E. , Flanagan, R. J. , & Karron, J. D. (2009). Ecology and evolution of plant‐pollinator interactions. Annals of Botany, 103, 1355–1363. 10.1093/aob/mcp122 19482881 PMC2701755

[ece34438-bib-0031] Newland, C. E. , & Wooller, R. D. (1985). Seasonal changes in a honeyeater assemblage in *Banksia* woodland near Perth, Western Australia. New Zealand Journal of Zoology, 12(4), 631–636. 10.1080/03014223.1985.10428312

[ece34438-bib-0032] Niedballa, J. , Sollmann, R. , Courtiol, A. , & Wilting, A. (2016). camtrapR: An R package for efficient camera trap data management. Methods in Ecology and Evolution, 7, 1457–1462 10.1111/2041-210X.12600

[ece34438-bib-0033] O'Connell, A. F. , Nichols, J. D. , & Karanth, K. U. (Eds.) (2011). Camera traps in animal ecology: Methods and analyses. New York, NY: Springer 10.1007/978-4-431-99495-4

[ece34438-bib-0034] Ollerton, J. , Alarcon, R. , Waser, N. M. , Price, M. , Watts, S. , Cranmer, L. , … Rottenberry, J. (2009). A global test of the pollination syndrome hypothesis. Annals of Botany, 103, 1471–1480. 10.1093/aob/mcp031 19218577 PMC2701765

[ece34438-bib-0035] Pauw, A. , & Stanaway, R. (2015). Unrivalled specialization in a pollination network from South Africa reveals that specialization increases with latitude only in the Southern Hemisphere. Journal of Biogeography, 42, 652–661. 10.1111/jbi.12453

[ece34438-bib-0036] Phillips, R. D. , Hopper, S. D. , & Dixon, K. W. (2010). Pollination ecology and the possible impacts of environmental change in the southwest Australian biodiversity hotspot. Philosophical Transactions of the Royal Society of London B, 365, 517–528. 10.1098/rstb.2009.0238 PMC2838264 20047877

[ece34438-bib-0037] Phillips, R. D. , Steinmeyer, F. , Menz, M. H. M. , Erickson, T. E. , & Dixon, K. W. (2014). Changes in the composition and behaviour of a pollinator guild with plant population size and the consequences for plant fecundity. Functional Ecology, 28, 846–856. 10.1111/1365-2435.12237

[ece34438-bib-0038] Potts, S. G. , Biesmeijer, J. C. , Kremen, C. , Neumann, P. , Schweiger, O. , & Kunin, W. E. (2010). Global pollinator declines: Trends, impacts and drivers. Trends in Ecology and Evolution, 25, 345–353. 10.1016/j.tree.2010.01.007 20188434

[ece34438-bib-0039] Proctor, M. , Yeo, P. , & Lack, A. (1996). The natural history of pollination. London, UK: Harper Collins.

[ece34438-bib-0040] Quintero, E. , Genzoni, E. , Mann, N. , Nuttman, C. , & Anderson, B. (2017). Sunbird surprise: A test of the predictive power of the syndrome concept. Flora, 232, 22–29. 10.1016/j.flora.2016.11.015

[ece34438-bib-0041] Ramsey, M. W. (1988a). Floret opening in *Banksia menziesii* R. Br.; the importance of nectarivorous birds. Australian Journal of Botany, 36, 225–232. 10.1071/BT9880225

[ece34438-bib-0042] Ramsey, M. W. (1988b). Differences in pollinator effectiveness of birds and insects visiting *Banksia menziesii* (Proteaceae). Oecologia, 76, 119–124. 10.1007/BF00379609 28312388

[ece34438-bib-0043] Ramsey, M. W. (1989). The seasonal abundance and foraging behaviour of honeyeaters and their potential role in the pollination of *Banksia menziesii* . Australian Journal of Ecology, 14, 33–40.

[ece34438-bib-0044] Ramsey, M. , & Vaughton, G. (1991). Self‐incompatibility, protandry, pollen production and pollen longevity in *Banksia menziesii* . Australian Journal of Botany, 39, 497–504. 10.1071/BT9910497

[ece34438-bib-0045] Regan, E. C. , Santini, L. , Ingwall‐King, L. , Hoffmann, M. , Rondinini, C. , Symes, A. , … Butchart, S. H. M. (2015). Global trends in the status of bird and mammal pollinators. Conservation Letters, 8, 397–403. 10.1111/conl.12162

[ece34438-bib-0046] Rosas‐Guerrero, V. , Aguilar, R. , Marten‐Rodriguez, S. , Ashworth, L. , Lopezaraiza‐Mikel, M. , Bastida, J. M. , & Quesada, M. (2014). A quantitative review of pollination syndromes: Do floral traits predict effective pollinators? Ecology Letters, 17, 388–400. 10.1111/ele.12224 24393294

[ece34438-bib-0047] Rovero, F. , Zimmermann, F. , Berzi, D. , & Meek, P. (2013). “Which camera trap type and how many do I need?” A review of camera features and study designs for a range of wildlife research applications. Hystrix, the Italian Journal of Mammalogy, 24, 148–156.

[ece34438-bib-0048] Steenweg, R. , Hebblewhite, M. , Kays, R. , Kays, R. , Ahumada, J. , Fisher, J. T. , … Rich, L. N. (2017). Scaling‐up camera traps: Monitoring the planet's biodiversity with networks of remote sensors. Frontiers in Ecology and the Environment, 15(1), 26–34. 10.1002/fee.1448

[ece34438-bib-0049] Tack, J. L. P. , West, B. S. , McGowan, C. P. , Ditchkoff, S. S. , Reeves, S. J. , Keever, A. C. , & Grand, J. B. (2016). AnimalFinder: A semi‐automated system for animal detection in time‐lapse camera trap images. Ecological Informatics, 36, 145–151. 10.1016/j.ecoinf.2016.11.003

[ece34438-bib-0050] Wiens, D. , Renfrew, M. , & Wooller, R. O. (1979). Pollen loads of honey possums (*Tarsipes spenserae*) and non flying mammal pollination in southwestern Australia. Annals of Missouri Botanic Garden, 66, 830–838. 10.2307/2398921

[ece34438-bib-0051] Wooller, R. , & Wooller, S. (2013). Sugar and sand: The world of the honey possum. Cottlesloe: Swanbrae Press.

[ece34438-bib-0052] Zoeller, K. C. , Steenhuisan, S. L. , Johnson, S. D. , & Midgley, J. J. (2016). New evidence for mammal pollination of *Protea* species (Proteaceae) based on remote camera analysis. Australian Journal of Botany, 64, 1–7. 10.1071/BT15111

